# Antioxidant Constituents of Hops (*Humulus lupulus* L.) as Functional Raw Materials for Cosmetic Applications

**DOI:** 10.3390/ma19040821

**Published:** 2026-02-21

**Authors:** Magdalena Dzienisik, Marta Marzec, Izabela Nowak

**Affiliations:** 1 Department of Applied Chemistry, Faculty of Chemistry, Adam Mickiewicz University in Poznań, Uniwersytetu Poznańskiego 8, 61-614 Poznań, Poland; magdzi9@amu.edu.pl; 2 BANDI Cosmetics Sp. z o.o., Warszawska 7, 05-152 Czosnów, Poland

**Keywords:** antioxidant, plant extract, cosmetics, ABTS assay, Folin–Ciocâlteu method, FRAP assay, anti-irritant properties

## Abstract

*Humulus lupulus* L. (hops) is best known for its application in the brewing industry; however, growing scientific interest has revealed its high content of natural antioxidants, including flavonoids and polyphenols. These compounds exhibit pronounced anti-inflammatory activity, positioning hops as a promising plant-derived ingredient for cosmetic use. The present study evaluates the antioxidant properties of *Humulus lupulus* L. (HL) extract and cosmetic formulations loaded with hop-based active substances. Antioxidant capacity was determined using ABTS, Folin–Ciocâlteu, and FRAP methods. The hop extract showed limited free radical scavenging activity and reducing power; however, these results still confirm its antioxidant potential. Importantly, cosmetic emulsions enriched with the HL extract maintained substantial antioxidant activity, demonstrating successful incorporation and stability of the bioactive compounds within the formulations. Physicochemical stability tests, including pH monitoring and visual evaluation during storage, indicated good stability under different conditions. In vivo studies confirmed the effectiveness of cosmetics containing HL extracts as natural anti-irritant agents, demonstrated by a 10% reduction in erythema after a three-week application period. These findings provide evidence supporting the use of *Humulus lupulus* L. in the development of plant-based cosmetic products designed to improve the health of skin prone to irritation.

## 1. Introduction

*Humulus lupulus* L. (common hop) is a perennial, climbing, dioecious plant belonging to the *Cannabaceae* family. While hops are widely associated with the brewing industry, they are also a well-known medicinal plant with a long-standing tradition of use in folk medicine. *Humulus lupulus* L. (HL) has been officially recognized as a medicinal plant by both the European Scientific Cooperative on Phytotherapy (ESCOP) and the Herbal Medicinal Product Committee (HMPC), based on extensive traditional use, particularly in the treatment of anxiety, sleep disorders, and mental restlessness [1,2,3]. *Humulus lupulus* L. is characterized by a rich chemical composition that includes a wide range of bioactive compounds exhibiting significant biological activity [2,4,5]. The concentration and ratio of these substances in the plant can vary and are influenced by multiple factors, such as hop variety, cultivation conditions, harvesting techniques, the degree of cone aging, and the specific climatic conditions in which the plant grows [6,7]. The active compounds found in hops can be classified into several major groups, each associated with specific biological activities. Among them, polyphenols, which constitute 4–14% of the dry matter of hop cones, have been extensively studied for their potent radical scavenging properties. Numerous reports emphasize that the antioxidant capacity of HL is primarily attributed to flavonoids, such as xanthohumol, isoxanthohumol, and 8-prenylnaringenin, as well as flavonols like quercetin and kaempferol glycosides [8]. What is more, they are primarily responsible for the anti-inflammatory effect [2,6]. Xanthohumol, the most abundant prenylchalcone in hops, has been documented to exhibit antioxidant activity significantly higher than that of vitamin E or α-tocopherol, owing to its ability to inhibit lipid peroxidation and neutralize reactive oxygen species (ROS) including superoxide anions and hydroxyl radicals [9]. The most important additional groups include: (a) volatile oil constituents, such as humulene and caryophyllene, which exhibit sedative and anti-inflammatory properties [7,10,11]; (b) bitter acids/resins, comprising α-acids (humulones) and β-acids (lupulones), which demonstrate antibacterial, antifungal, and antiviral activities [4,10,12]. It is worth noting that while bitter acids are primarily known for their preservative and flavoring properties, the recent literature also highlights their contribution to the overall antioxidant pool of the plant, particularly through the chelation of transition metal ions and the inhibition of lipid peroxidation [13]. Moreover, the content of α-acids is considered one of the most important quality parameters of hops, as these compounds are primarily responsible for the characteristic bitterness of the plant [4,12]. These compounds can activate TAS2R-type bitter taste receptors expressed in skin cells, including keratinocytes and fibroblasts [14]. Recent evidence suggests that bitter taste receptors in the skin contribute to cutaneous defense mechanisms, such as antibacterial and anti-inflammatory responses, and may play a role in modulating immune activity in response to environmental chemical stimuli [15,16,17].

Hop extract exhibits a range of biologically significant properties that may positively influence the condition and function of the skin. Its documented soothing, anti-inflammatory, and skin-reactivity-reducing effects make it a particularly valuable ingredient in cosmetic formulations designed for sensitive and hyperreactive skin [4,14,18]. The antioxidant potential of hops is of particular interest in dermatology, as it has been shown to protect skin cells from oxidative stress induced by UV radiation and environmental pollutants, thereby preventing premature aging and collagen degradation [19,20]. A clinical *in vivo* study demonstrated that *Humulus lupulus* L. extract, rich in bitter acids (humulone and lupulone) and obtained via supercritical CO_2_ extraction, exhibits significant anti-inflammatory activity when applied topically in an oil-in-water (O/W) cream. However, the same extract in a water-in-oil (W/O) emulsion failed to reduce UVB-induced erythema, indicating that the anti-inflammatory efficacy of the extract depends not only on its bioactive compounds but also critically on the type of topical formulation [21]. Therefore, establishing a formulation system that ensures both physicochemical stability and retention of biological activity is essential for the successful incorporation of hops into skin care products. Growing consumer interest in natural bioactive ingredients supporting skin barrier function and reducing irritation highlights the need for new plant-derived actives with multifunctional properties. Hops represent a promising candidate due to the combination of soothing effects and strong antioxidant defense against reactive oxygen species—a major contributor to skin aging and inflammatory disorders [14,18]. Furthermore, the industry lacks comprehensive studies linking the biological properties of hop extract with its formulation behavior in finished cosmetic products, which is crucial for ensuring functional efficacy on the skin.

The objective of the presented study was to comprehensively evaluate the antioxidant properties of hop extract and to develop and characterize an O/W cosmetic emulsion containing this extract, with particular emphasis on its physicochemical parameters, antioxidant potential, and anti-irritant efficacy evaluated using in vivo methods. This publication is distinctive because it integrates two areas that are often treated separately: a comprehensive, multi-parameter evaluation of the antioxidant capacity of hop extract and its direct translation into a finished O/W cosmetic emulsion. Its novelty lies in moving beyond a “proof-of-activity” approach for the raw extract and demonstrating how incorporation into a real formulation affects key physicochemical attributes (e.g., pH, texture profile, stability), while simultaneously verifying whether the antioxidant potential is retained within the emulsion matrix (non-invasive methods of skin testing). In this way, the study provides practical, formulation-relevant evidence that supports rational design of effective and stable plant-based cosmetics, rather than limiting conclusions to in vitro antioxidant screening alone. Additionally, to the best of our knowledge, FRAP data (expressed in µmol TE/g) for active substances incorporated into O/W cosmetic emulsions have not been previously reported, which makes our study the first to provide this type of formulation-relevant reducing power assessment.

## 2. Materials and Methods

### 2.1. Humulus Lupulus L. Extract

In this study, a commercial extract of *Humulus lupulus* L. was used—trade name: Senseryn™ (Provital, S.A., Barcelona, Spain); INCI: Propanediol, Glycerin, *Humulus Lupulus* Extract, Citric Acid. The raw material is obtained through solvent extraction from the cones of the Cascade hop variety, one of the most commonly used in craft production. Senseryn™ is a transparent to slightly turbid solution (density: 1.070–1.150 g/mL), with a light brown to brown color and a characteristic odor typical of aqueous-glycolic plant extracts. The extract is standardized for total polyphenol content (0.10–0.30%) [22] and complies with the requirements for cosmetic ingredients as defined by Regulation (EC) No 1223/2009 [23]. In accordance with ISO 16128, the extract contains 100% ingredients of natural origin [24].

#### Qualitative Analysis Using High-Performance Liquid Chromatography (HPLC)

Chromatographic analysis was performed using a Varian LC-940 liquid chromatograph (Varian, Palo Alto, CA, USA) equipped with a diode array detector (DAD). The separation was carried out on a Kromasil 100 column (C18; 250 × 4.6 mm, 5 µm; Nouryon AB, Gothenburg, Sweden) thermostated at 30 °C. The mobile phase consisted of (A) an aqueous phosphoric acid solution (0.085 wt.%; TCI Chemicals, Zwijndrecht, Belgium) and (B) acetonitrile (Merck, Darmstadt, Germany), eluted according to the gradient conditions specified in Table 1.

The total analysis time was 50 min, with a flow rate of 1.0 mL/min and an injection volume of 10 µL. Detection was performed at a wavelength of 370 nm. Sample preparation involved dissolving Senserin™ and xanthohumol (XAntiAge™, A-Sense, Poniatowa, Poland) in methanol to obtain concentrations of 0.2 and 0.02 mg/mL, respectively. The resulting solutions were filtered through 0.45 µm membrane filters and transferred into autosampler vials. Samples were stored at 4 °C until analysis.

### 2.2. Formulation Strategies for O/W Emulsions Designed for Sensitive Skin Care

#### 2.2.1. Technological Process Description for O/W Emulsions Formulated with and Without HL Extract

**Figure 1:**
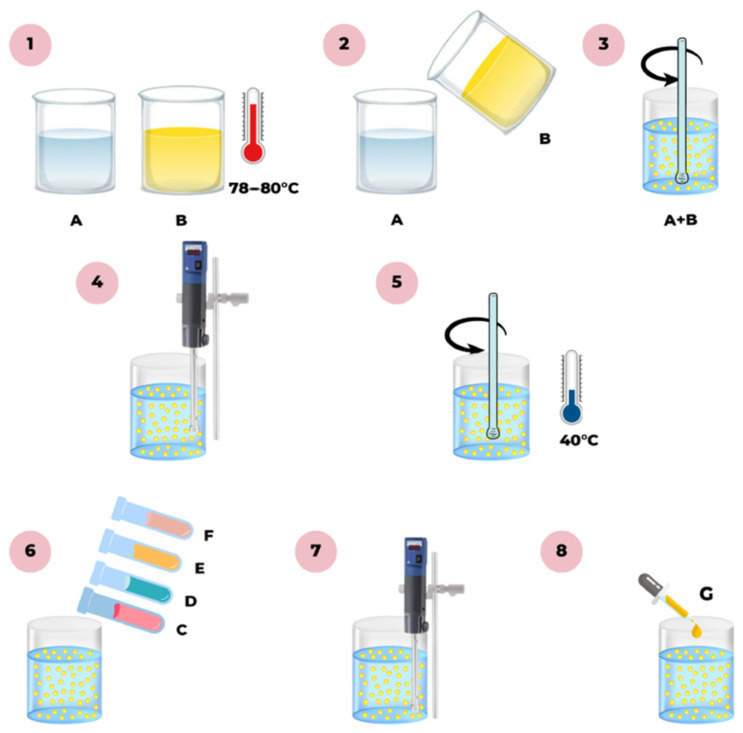
Simplified schematic of the laboratory-scale formulation process for the developed O/W emulsion. Raw material identifications: (A) rheology modifiers; (B) emulsifiers, emollients, and stabilizers; (C–F) active ingredients; and (G) functional ingredients.

The formulation process began with the development of preliminary prototypes of O/W emulsions, prepared in accordance with a proprietary technological methodology. The preparation stages included, sequentially: weighing of raw materials, heating the individual emulsion phases to a temperature of 80–85 °C, combining the phases, homogenization, mixing, and finally controlled cooling (Figure 1). The detailed technological procedure constitutes proprietary knowledge (know-how) of BANDI Cosmetics Sp. z o.o. (Czosnów, Poland).

By modifying the composition, type, and percentage content of individual raw materials in successive trials, the most optimal formulation was achieved. The final version of the emulsion formula, containing 3.0 wt.% of HL extract, was designated with the code DW01. For comparative study purposes, a reference emulsion without the extract was also prepared (code DW02), in which the HL extract was replaced with 3.0 wt.% of water. The selection of the chosen extract concentration was based on preliminary formulation screening, which demonstrated that this concentration provided the most favorable balance between biological activity—ensuring the intended cosmetic functionality—and acceptable sensory properties of the emulsion (appearance, consistency, color, and odor).

#### 2.2.2. Qualitative Composition of the Designed Emulsions

As the next step, the compositions of the designed O/W emulsions were developed in accordance with the International Nomenclature of Cosmetic Ingredients (INCI), in line with Article 19 of Regulation (EC) No 1223/2009 on cosmetic products. The formulations of emulsions DW01 and DW02 are presented below, including the percentage ranges of individual ingredients, as required by the Cosmetic Products Notification Portal (CPNP): (A) 75–100 wt.%; (B) 50–75 wt.%; (C) 25–50 wt.%; (D) 10–25 wt.%; (E) 5–10 wt.%; (F) 1–5 wt.%; (G) 0.1–1 wt.%; (H) 0–0.1 wt.%.

DW01—O/W Emulsion containing 3.0 wt.% of HL extract.

Ingredients (INCI): Aqua/Water (B), Coco-Caprylate/Caprate (F), Isostearyl Isostearate (F), Propanediol (F), Trehalose (F), Tripelargonin (F), Arachidyl Alcohol (F), Glycerin (F), Glyceryl Stearate (F), Pentaerythrityl Distearate (G), Pentylene Glycol (G), Butylene Glycol (G), Humulus Lupulus (Hops) Extract (H), Ceramide NP (G), Carnosine (G), Hydroxyphenyl Propamidobenzoic Acid (G), Saccharomyces/Acetobacter/Camellia Sinensis Leaf Extract Ferment Filtrate (G), Plukenetia Volubilis Seed Oil (G), Olea Europaea (Olive) Fruit Oil (G), Tocopherol (H), Behenyl Alcohol (G), Stearic Acid (G), Arachidyl Glucoside (G), Lecithin (G), Sodium Acrylates Copolymer (G), Hydroxypropyl Starch Phosphate (H), Polyglyceryl-10 Laurate (H), Hydroxyacetophenone (G), Caprylyl Glycol (G), Citric Acid (H), Ascorbyl Palmitate (H), and 1,2-Hexanediol (G).

DW02—O/W Emulsion without HL extract.

Ingredients (INCI): Aqua/Water (B), Coco-Caprylate/Caprate (F), Isostearyl Isostearate (F), Propanediol (F), Trehalose (F), Tripelargonin (F), Arachidyl Alcohol (F), Glyceryl Stearate (F), Pentaerythrityl Distearate (G), Pentylene Glycol (G), Glycerin (G), Butylene Glycol (G), Ceramide NP (G), Carnosine (G), Hydroxyphenyl Propamidobenzoic Acid (G), Saccharomyces/Acetobacter/Camellia Sinensis Leaf Extract Ferment Filtrate (G), Plukenetia Volubilis Seed Oil (G), Olea Europaea (Olive) Fruit Oil (G), Tocopherol (H), Behenyl Alcohol (G), Stearic Acid (G), Arachidyl Glucoside (G), Lecithin (G), Sodium Acrylates Copolymer (G), Hydroxypropyl Starch Phosphate (H), Polyglyceryl-10 Laurate (H), Hydroxyacetophenone (G), Caprylyl Glycol (G), Citric Acid (H), Ascorbyl Palmitate (H), and 1,2-Hexanediol (G).

#### 2.2.3. Aging Tests of the Formulated Cosmetic Products

The DW01 and DW02 emulsions were subjected to aging studies. The emulsion samples were stored at three different temperatures for a period of three months: room temperature (RT), 4 °C, and 45 °C. The samples were evaluated organoleptically (appearance, odor, color, consistency) and using a pH-meter CP-411 (Elmetron, Zabrze, Poland).

### 2.3. Evaluation of the Antioxidant Properties of the Hop Extract, Xanthohumol, and the O/W Emulsion Containing It

The antioxidant activity of the hop extract was evaluated for methanolic solutions of Senseryn™ (at concentrations of 1.0 wt.%, 2.0 wt.%, and 3.0 wt.%) and pure xanthohumol (XAntiAge™, A-Sense, Poniatowa, Poland) at concentrations of 0.1 wt.%, 0.2 wt.%, and 0.3 wt.%, reflecting the natural abundance of xanthohumol in HL extract. In addition, the developed emulsion DW01 containing hop extract was also subjected to testing. A methanolic solution of the investigated emulsion was prepared at a concentration corresponding to the declared level of efficacy and the potential effectiveness of the extract in the applied cosmetic formulation.

To assess the antioxidant activity of the hop extract, xanthohumol, and the HL extract-enriched emulsion, three spectrophotometric methods were employed: the ABTS assay, the FRAP assay, and the Folin–Ciocâlteu method. The analyses were performed using a UV-Vis spectrophotometer Cary 50 (Varian, Palo Alto, CA, USA).

#### 2.3.1. Determination of Antioxidant Activity Using the ABTS Method

The first stage of the procedure involved the generation of the ABTS radical cation, starting with the separate preparation of an ABTS solution (2,2′-azino-bis(3-ethylbenzothiazoline-6-sulfonic acid; Merck, Darmstadt, Germany) at a concentration of 7.4 mmol/dm^3^ and a potassium persulfate solution (Chempur, Piekary Śląskie, Poland) at a concentration of 2.6 mmol/dm^3^. The obtained solutions were then mixed in a 1:1 volume ratio and left in the dark for 16 h. Subsequently, the mixture was diluted with methanol (Chempur, Piekary Śląskie, Poland) until the absorbance of the solution reached approximately 0.7 (λ = 734 nm). For the preparation of the calibration curve, methanolic solutions of Trolox (6-hydroxy-2,5,7,8-tetramethylchroman-2-carboxylic acid; TCI Chemicals, Zwijndrecht, Belgium) were prepared at concentrations of 0.0125, 0.025, 0.05, 0.075, 0.1, 0.15, and 0.2 mmol/dm^3^. Subsequently, 1.5 mL of each Trolox solution and 2.8 mL of the ABTS^•+^ solution were added to cuvettes. The cuvettes were incubated at room temperature in the dark for 6 min, after which absorbance was measured at λ = 734 nm. In the final stage of the described procedure, previously prepared methanolic solutions of HL extract, xanthohumol, and DW01 emulsion were filtered through 0.45 μm membrane filters, and 0.15 mL of each filtrate was added to separate cuvettes containing 2.8 mL of the ABTS cation radical solution. The cuvettes were incubated at room temperature in the dark for 6 min, followed by absorbance measurement at λ = 734 nm. All absorbance measurements were performed in triplicate, and the results are presented as mean values. Antioxidant activity was expressed as the Trolox Equivalent Antioxidant Capacity (TEAC) and as the percentage of inhibition.

#### 2.3.2. Determination of Total Phenolic Content Using the Folin–Ciocâlteu Method

The Folin–Ciocâlteu method procedure involved the use of a ready-to-use Folin–Ciocâlteu reagent (a mixture of sodium tungstate, sodium molybdate, lithium sulfate, bromine water, and concentrated hydrochloric and phosphoric acids; Chempur, Piekary Śląskie, Poland), which was diluted tenfold before use. Simultaneously, a sodium carbonate solution with a concentration of 2.26 mol/dm^3^ was prepared (Pol-Aura, Zawroty, Poland). For the preparation of the calibration curve, methanolic solutions of gallic acid (Pol-Aura, Zawroty, Poland) were prepared at concentrations of 5, 15, 25, 50, 100, 150, 200, and 300 mg/dm^3^. Subsequently, 0.1 mL of each gallic acid solution, 0.5 mL of sodium carbonate solution (Merck, Darmstadt, Germany), and 2.5 mL of the Folin–Ciocâlteu reagent were added to cuvettes. The cuvettes were incubated at room temperature in the dark for 2 h, and their absorbance was then measured at λ = 760 nm. In the final stage of the described procedure, previously prepared methanolic solutions of the HL extract, xanthohumol, and DW01 emulsion were filtered through 0.45 μm membrane filters. Then, 0.1 mL of each filtrate was added to separate cuvettes containing 2.5 mL of the Folin–Ciocâlteu reagent. The cuvettes were incubated at room temperature in the dark for 2 h, after which 0.5 mL of the sodium carbonate solution was added, and the absorbance was measured immediately after mixing at λ = 760 nm. All absorbance measurements were performed in triplicate, and the results are presented as mean values. The total phenolic content (TPC) was expressed as gallic acid equivalents (GAEs).

#### 2.3.3. Determination of Antioxidant Activity Using the FRAP Method

The first stage of the procedure involved the preparation of the FRAP reagent, beginning with the separate preparation of a hydrochloric acid solution (Merck, Darmstadt, Germany) at a concentration of 40 mmol/dm^3^, to which 0.0755 g of TPTZ (2,4,6-tripyridyl-s-triazine; Pol-Aura, Zawroty, Poland) was added. Subsequently, a FeCl_3_·6H_2_O solution (Chempur, Piekary Śląskie, Poland) with a concentration of 20 mmol/dm^3^ and an acetate buffer at pH 3.6 (CPAChem, Bogomilovo, Bulgaria) were prepared. The obtained solutions were then mixed in a volumetric ratio of 10:1:1 (buffer:TPTZ in HCl:FeCl_3_·6H_2_O) and heated to 37 °C until a uniform color developed. The mixture was then diluted until the absorbance reached approximately 0.7 (λ = 595 nm). For the preparation of the calibration curve, methanolic solutions of Trolox were prepared at concentrations of 0.015, 0.1, 0.2, 0.5, 1.0, 1.5, and 2.0 mmol/dm^3^. Subsequently, 0.1 mL of each Trolox solution and 3.9 mL of the FRAP reagent were added to cuvettes. The cuvettes were incubated at 37 °C for 30 min, and absorbance was measured at λ = 595 nm. In the final stage of the described procedure, previously prepared methanolic solutions of the HL extract, xanthohumol, and DW01 emulsion were filtered through 0.45 μm membrane filters. Then, 0.1 mL of each filtrate was added to separate cuvettes containing 3.9 mL of the FRAP reagent. The cuvettes were incubated at 37 °C for 30 min, followed by absorbance measurement at λ = 595 nm. All absorbance measurements were performed in triplicate, and the results are presented as mean values. The antioxidant activity (ferric ion reducing capacity) was expressed as Trolox equivalent antioxidant capacity (TEAC).

### 2.4. In Vivo Skin Response to a Hop Extract-Enriched Emulsion After Induced Irritation

#### 2.4.1. Panel of Participants

The study included 16 volunteers (aged 23–62 years) with skin prone to irritation. All participants were informed in detail about the study objectives, procedures, potential risks, and benefits before enrollment. Written informed consent for participation in the study was obtained from all volunteers. In addition, consent for the processing of personal data was collected in accordance with applicable data protection regulations. Participants received written information materials describing the study protocol and were allowed to ask questions before and during the study. Volunteers were free to withdraw from the study at any time without providing a reason and without any consequences.

#### 2.4.2. In Vivo Study Protocol

**Figure 2:**
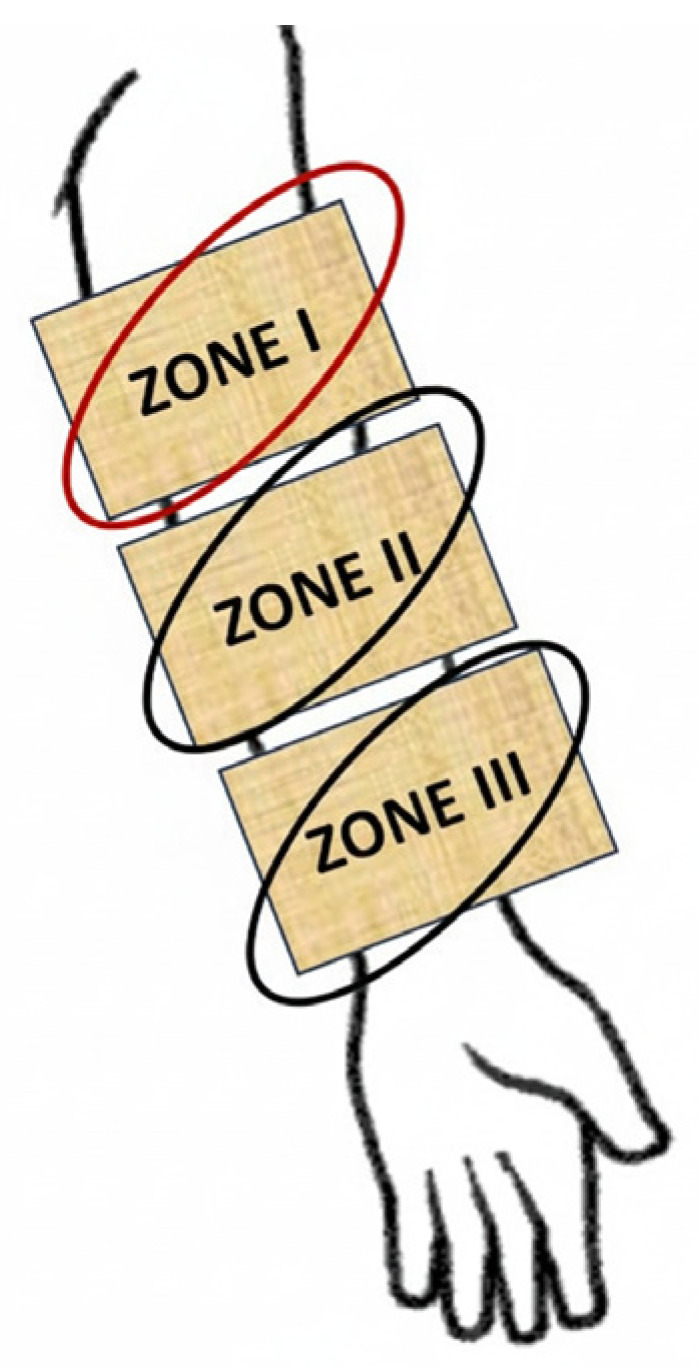
Schematic representation of the application of formulations DW01 and DW02 on the left forearm.

The study protocol comprised two consecutive phases: (I) an irritation phase (week 0–week 1, W0–W1) and (II) a regeneration phase (week 1–week 3, W1–W3). To evaluate the potential regenerative effects of formulations DW01 and DW02, a controlled disruption of the epidermal skin barrier was induced during the first phase of the study (W0–W1, irritation phase) using a 5.0 wt.% solution of Sodium Laureth Sulfate (SLES, an anionic surfactant commonly used as a skin barrier-disrupting agent). Volunteers washed the skin of the entire left forearm with the SLES-containing formulation twice daily (morning and evening) for one week. Following completion of the 7-day irritation phase, a 3-week care/regeneration phase was initiated on the skin of the left forearm. During this phase, cosmetic emulsions were applied according to the scheme provided in the study materials (Figure 2): zone I—emulsion DW01 (containing hop extract); zone II—base emulsion DW02; and zone III (control)—an untreated area with no care intervention.

#### 2.4.3. Non-Invasive Instruments for Skin Assessment

All procedures were non-invasive and performed under controlled conditions to ensure participant safety and comfort. Measurements of selected skin parameters were carried out at weeks 0, 1, 2, and 3 on areas of the left forearm in all designated zones (I, II, III). The study employed the Courage + Khazaka apparatus set (Courage + Khazaka electronic GmbH, Cologne, Germany), including the following instruments: Corneometer^®®^ CM 825—to assess stratum corneum hydration; Tewameterr^®®^ TM 300—to determine transepidermal water loss (TEWL); Mexameter^®®^ MX 18—to quantify erythema levels (skin redness). For each parameter, measurements were repeated three times, and the arithmetic mean and standard deviation were calculated. Statistical analyses were performed using Statistica 13.3 software (StatSoft, Cracow, Poland). The significance of changes in skin parameter values over time was evaluated using the Kruskal–Wallis test, while differences between application zones (corresponding to the applied products) were assessed using ANOVA. A significance level of *p* < 0.05 was adopted for all tests.

## 3. Results and Discussion

### 3.1. Characterization of Hop Extract by Liquid Chromatography

**Figure 3:**
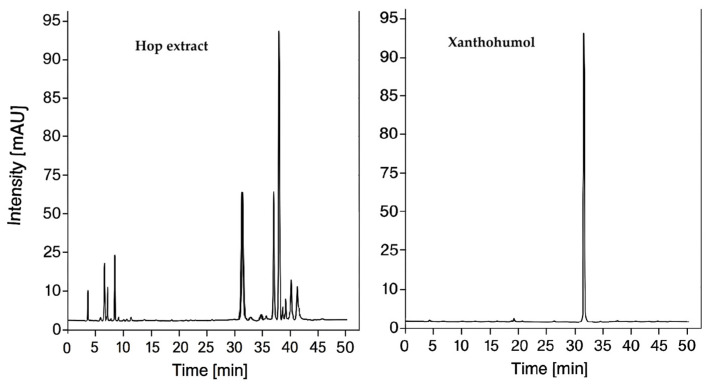
Chromatograms of the hop extract (**left**) and the xanthohumol standard (**right**).

Analysis of the hop extract by HPLC-DAD allowed the separation and identification of the main compounds belonging to soft and hard resins, including α- and β-acids, humulinones, and chalcone derivatives (xanthohumol and isoxanthohumol). Based on retention times (t_R_), the detected peaks (Figure 3) were correlated with reference standards (xanthohumol) and literature data, which enabled confirmation of the identity of the hop-derived compounds. In the initial part of the chromatogram (6–10 min), humulinones and iso-α-acids were observed, whereas at later retention times (30–40 min), chalcone derivatives (xanthohumol and isoxanthohumol) as well as α- and β-acids were detected. This elution pattern was consistent with the expected separation in the applied chromatographic system, progressing from more polar to less polar compounds.

Comparison of the obtained retention times with those reported in the literature showed good agreement for humulinones and iso-α-acids, whereas higher t_R_ values were observed for xanthohumol and isoxanthohumol (Table 2). The prolonged retention of these compounds may be attributed to the use of a milder gradient, an extended analysis time, and somewhat different separation conditions. A similar effect was described by Duarte et al. [25], who reported that a higher proportion of water in the initial mobile phase, combined with an increased share of the organic phase in the final stage of elution, resulted in a delayed elution of less polar compounds. To confirm the presence of xanthohumol—a bioactive compound of particular interest in the context of this study due to its antioxidant properties—an additional HPLC analysis was performed under identical chromatographic conditions using a xanthohumol reference standard. The obtained retention time of 31.789 min correlated well with the experimentally determined value (t_R_ = 31.621 min). Moreover, the chromatographic separation enabled clear resolution of all major groups of hop-derived compounds. The distinct separation of α- and β-acids (t_R_ = 38.638 and 39.684 min, respectively) confirmed the high selectivity of the applied C18 column and the suitability of the extended gradient, which markedly increased the proportion of the organic phase in the later stages of elution. The resulting qualitative profile of the HL extract demonstrated the dominance of α-acids as well as the presence of considerable amounts of xanthohumol and its isomers. This composition corresponds to typical methanolic hop extracts and may indicate a notable antioxidant potential of the analyzed raw material (see Section 3.3, concerning the assessment of the antioxidant activity of the HL extract).

### 3.2. Accelerated Aging Tests of the Developed Cosmetic Products

Stability assessment of cosmetic emulsions under varying temperature conditions is a standard and essential practice in evaluating the quality and shelf life of cosmetic products. To investigate the effect of hop extract on the quality and stability of O/W emulsions, both the DW01 emulsion (Table 3) and a reference emulsion DW02 without the extract (Table 4) were subjected to testing. Accelerated aging conditions (temperature of 45 °C) were applied to simulate several months of product aging within a significantly shorter time frame. This approach made it possible to estimate whether the emulsion is likely to maintain its properties throughout the declared shelf life [30]. Stability testing is part of the mandatory documentation (the so-called cosmetic product safety report) in accordance with Regulation (EC) No. 1223/2009 [23].

**Figure 4:**
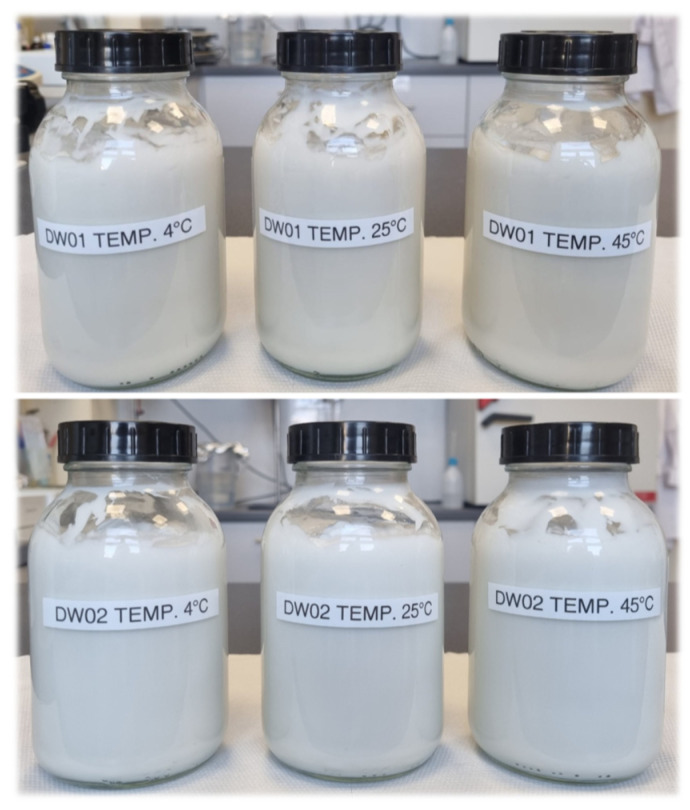
Samples of the tested cosmetic emulsions DW01 (**top**) and DW02 (**bottom**) after completion of the accelerated stability test.

A comparative design of aging tests for O/W emulsions, both containing and lacking hop extract, enabled the evaluation of the extract’s impact on the stability and overall physicochemical properties of the final cosmetic product (Figure 4).

It was verified whether the addition of HL extract causes destabilization (e.g., phase separation, product turbidity) or, conversely, stabilizes the emulsion system. The tested hop extract contains a range of bioactive compounds that may degrade over time, especially at elevated temperatures of 45 °C, potentially leading to color changes (e.g., browning), sediment formation, decreased stability, and noticeable shifts in pH values [31]. The observed color and odor changes in the DW01 emulsion at 45 °C are a result of the thermal reactivity of natural bioactive compounds present in the HL extract (Figure 4, top), which is typical for plant-derived extracts [32,33]. These changes were organoleptic in nature and did not cause technological destabilization of the emulsion system. The absence of significant differences in other aspects (no phase separation, stable pH values ranging from 5.5 to 6) confirmed the lack of negative impact of the extract on the physicochemical integrity of the product and its compatibility with the developed O/W emulsion system. The formulated DW01 emulsion was properly developed in terms of physicochemical, functional, and stability parameters. The sensory and application profile of the product was determined to be optimal for cosmetic formulations intended for skin application (face cream), indicating that it can be safely used in skincare, including sensitive and irritation-prone skin.

### 3.3. Antioxidant Properties of the Hop Extract, Xanthohumol, and the HL-Enriched Emulsion

The assessment of antioxidant activity most commonly involves spectrophotometric methods, which are primarily based on the ability of the tested chemical substances to scavenge free radicals or reduce metal ions. In the present study, for a comprehensive evaluation of the antioxidant activity of hop extract, xanthohumol, and the HL-enriched emulsion, the following methods were employed:

- ABTS test—determining the ability to neutralize the ABTS radical (2,2′-azino-bis(3-ethylbenzothiazoline-6-sulfonic acid)); calibration curve equation: y = −1.5473x + 0.5875; R^2^ = 0.9961;
- Folin–Ciocâlteu method—used for the determination of total phenolic content; calibration curve equation: y = 0.0049x + 0.0142; R^2^ = 0.9992;
- FRAP test (Ferric Reducing Antioxidant Power)—allowing the evaluation of the sample’s capacity to reduce Fe^3+^ ions to Fe^2+^ ions; calibration curve equation: y = 1.0195x + 0.1829; R^2^ = 0.9937.

#### 3.3.1. ABTS Assay

The principle of the ABTS method is based on the reaction of the cation radical (ABTS^+•^), which exhibits an intense blue-green color and is formed by the oxidation of ABTS with potassium persulfate. Upon addition of an antioxidant to the radical solution, neutralization occurs, resulting in a decrease in absorbance at 734 nm, observed as a fading of the solution’s color [34]. The ABTS method is considered reliable for assessing the antioxidant properties of plant materials due to its ability to accurately reflect the capacity of the tested substances to scavenge both hydrophilic and lipophilic free radicals [34]. The ABTS assay results obtained for hop extract and xanthohumol are summarized in Table 5.

No linear correlation was observed between the ability to scavenge the ABTS radical and the concentration of the tested hop extract samples. Although inhibition increased with extract concentration (24–63%), the TEAC values (expressed in µmol TE/g) did not show strict proportionality. This effect results from normalization of antioxidant capacity per gram of sample and from approaching the upper linear response range of the ABTS assay at higher concentrations, where radical scavenging kinetics and partial saturation may influence the calculated values. The TEAC values obtained for the tested hop extract (3.3–17.4 µmol TE/g) confirm a detectable but limited capacity of the extract to directly scavenge free radicals in model chemical systems. The relatively low antioxidant activity of the tested extract may be attributed either to a reduced content of active compounds in the commercial extract or to the presence of specific fractions with selective biological activity, which may not be fully captured by the ABTS assay. Moreover, correlating literature-reported TEAC values for selected hop extracts (HL) (ranging from 0.11 to 8.26 µmol TE/g) [35,36] with the values obtained experimentally in this study showed very close agreement (3.3–17.4 µmol TE/g). However, the observed differences between experimental and literature data may have been influenced by several factors, including: (a) the extraction method—the type of solvent and extraction conditions significantly affect the phenolic content of the extract; (b) the plant part used for extraction—hop cones differ in antioxidant content depending on the stage of maturation; and (c) cultivation and storage conditions—environmental factors and storage practices determine the levels of biologically active compounds [6,37,38]. In the case of xanthohumol, a bioactive compound with a broad spectrum of activity, a correlation was observed between its ability to scavenge the ABTS radical and the concentration of the active substance in the tested samples. Xanthohumol exhibited a noticeable, dose-dependent antioxidant activity, reflected by increases in TEAC values and inhibition with rising concentrations of the tested solutions. The highest measured TEAC value (45.3 µmol TE/g), recorded at the maximum concentration of 0.3 wt.%, confirms the moderate capacity of the active compound to scavenge free radicals. In a study by Zhang et al., the antioxidant activity of xanthohumol was evaluated using the ABTS assays, yielding TEAC values of 0.32 ± 0.09 µmol TE/L, indicating moderate antioxidant potential compared to classical antioxidants like trolox, quercetin, and rutin [39].

#### 3.3.2. Folin–Ciocâlteu Test

The principle of the Folin–Ciocâlteu method is based on the reaction between the Folin–Ciocâlteu reagent and phenolic compounds present in the sample, leading to the formation of a blue complex whose color intensity is measured spectrophotometrically at 760 nm [40]. The results of the Folin–Ciocâlteu assay obtained for the hop extract and xanthohumol are presented in Table 6.

The total phenolic content (TPC) of the investigated hop extract was determined to be in the range of 0.81–0.99 mg GAE/g, which can be interpreted as a medium level. It was observed that TPC values tended to decrease with increasing extract concentration, even though the absorbance of the solutions increased. This phenomenon may be attributed to the nature of TPC calculations, where at higher sample concentrations, absorbance does not increase proportionally to the phenolic content, resulting in the observed decrease in TPC expressed per gram of sample. This non-linear response at higher concentrations is a documented limitation of the Folin–Ciocâlteu assay, as absorbance tends to deviate from linearity beyond the optimal range, and similar trends have been reported in other plant extracts [41].

The measured values, however, do not deviate significantly from literature data for selected hop extracts, although they tend to fall within the lower end of the reported range, where TPC values typically range from 0.51 to 6.60 mg GAE/g (Table 7). This discrepancy may result from differences in the extraction method (especially the solvent), the degree of sample dilution, the plant material used (plant variety), and the previously mentioned limitation of the linear range of the Folin–Ciocâlteu method at high sample concentrations.

Moving on to xanthohumol, the determined TPC values were considerably higher (8.08–11.93 mg GAE/g) compared to the HL extracts. However, xanthohumol represents a single phenolic compound, and due to its high bioactivity, it largely contributes to the phenolic activity of the HL extract. It should be noted, however, that the Folin–Ciocâlteu is a nonspecific assay that reflects the total phenolic content but does not directly assess their biological activity [46], which may be a limitation in the case of xanthohumol. Nevertheless, this method provides a complementary assessment alongside ABTS and FRAP assays, and given the presence of phenolic compounds in the tested extract, the obtained results represent a meaningful indicator of its antioxidant potential [43].

#### 3.3.3. FRAP Assay

The FRAP assay (Ferric Reducing Antioxidant Power) is based on the ability of antioxidants to reduce ferric ions (Fe^3+^) to ferrous ions (Fe^2+^). The resulting Fe^2+^ ions form a colored complex with TPTZ (2,4,6-tris(2-pyridyl)-s-triazine), the absorbance intensity of which is measured spectrophotometrically at 595 nm [47]. The results of the FRAP assay obtained for the hop extract and xanthohumol are summarized in Table 8.

The results obtained using the FRAP method for the hop extract (HL) indicate a limited reducing capacity (Fe^3+^ to Fe^2+^), even at higher sample concentrations. However, the measured values increased proportionally with the sample concentration, demonstrating a clear correlation with the increase in antioxidant activity. The FRAP values obtained in this assay (7.90–9.71 μmol TE/g) can be considered relatively low compared to the literature data for selected HL extracts, where FRAP values most commonly range from 60.0 to 75.0 μmol TE/g [48]. For xanthohumol, a similar trend was observed—the reducing capacity of the samples was directly proportional to the concentration of the tested solutions, consistent with the expected activity of the compound under investigation. An exception was noted for the xanthohumol sample at a 0.3 wt.% concentration, for which the measured value (209.5 μmol TE/g) was unexpectedly lower than that of the 0.2. wt.% sample (239.3 μmol TE/g). This discrepancy may be explained by the fact that the FRAP method exhibits a linear response only up to a certain absorbance value (approximately 0.8), and above this threshold (0.8240 in the case of the outlier sample), the reaction ceases to be proportional to the antioxidant concentration, as the TPTZ reagent is largely reduced. Nevertheless, the FRAP values for xanthohumol were significantly higher than those for the HL extract, likely due to the substantially greater reducing capacity of pure xanthohumol. Additionally, it is important to mention a limitation of the FRAP method in the context of assessing plant materials: due to the specific reaction conditions (acidic environment), some antioxidant compounds, such as those containing thiol groups, may not be fully detectable or quantifiable [47,49].

#### 3.3.4. Antioxidant Activity of the O/W Emulsion Containing Hop Extract

To assess the impact of the cosmetic medium—the emulsion—on the antioxidant activity of a formulation containing 3.0 wt.% of hop extract (DW01), analogous measurements of antioxidant activity were performed using the ABTS, FRAP, and Folin–Ciocâlteu assays. In addition, the control formulation (DW02, without extract) was tested, and its antioxidant activity was found to be practically undetectable. The obtained results are summarized in Table 9.

The antioxidant activity of the developed O/W emulsion enriched with hop extract was confirmed—the finished cosmetic product exhibited significantly stronger antioxidant properties compared to the tested HL extract. Such an improvement is frequently observed in cosmetic systems and is associated with formulation-related mechanisms strengthening the functional expression of active ingredients. This effect can be attributed to the synergistic action of Senseryn™ together with other emulsion components, such as tocopherol and carnosine. Additionally, emulsification provides protective effects toward phenolic compounds, limiting oxidation, volatilization, and photodegradation, which further enhances their reactivity in the finished formulation. The enhanced antioxidant activity may result from improved stabilization and bioavailability of the HL extract within the O/W emulsion matrix. The relatively positive TEAC and FRAP result (16.2 and 68.3 µmol TE/g, respectively) indicate the presence of effective concentrations of bioactive compounds with antioxidant properties in the product formulation. These compounds are capable of neutralizing reactive oxygen species (ROS), thereby reducing potential oxidative damage to skin cells. This suggests a potential contribution of the formulation to skin-protective effects, as reported in dermocosmetic applications of natural polyphenols. Based on the obtained results, it can be concluded that the designed hop extract-containing emulsion represents a stable and effective cosmetic form, potentially supporting the protection of the skin against oxidative stress. This conclusion is additionally supported by the favorable stability profile of the product demonstrated during the three-month testing period under different storage conditions. These findings are consistent with literature data on antioxidant cosmetic products (Table 9). Thus, the results confirm that HL extract is a promising natural ingredient for formulations aimed at combating oxidative stress in the skin. Overall, the combined TEAC–FRAP–TPC dataset provides an unusually complete and practically relevant antioxidant characterization that helps differentiate our extract from those previously reported and strengthens its justification as a functional cosmetic active.

### 3.4. Effects of an HL-Enriched Emulsion on Skin Barrier Recovery

#### 3.4.1. Irritation Phase (W0–W1)

A skin irritation induction procedure using a Sodium Laureth Sulfate (SLES) solution was performed to evaluate the skin response under conditions of increased sensitivity. After 7 days (W1) of SLES solution application on the left forearm, an increase in transepidermal water loss (TEWL) was observed in all measurement zones, specifically by 37% (zone I, *p* = 0.002), 36% (zone II, *p* = 0.003), and 33% (zone III, *p* = 0.003) compared to baseline values at the start of the study (W0). Skin hydration showed a slight increase of 8%, 6%, and 2% across zones I, II, and III, respectively, relative to week 0. However, these changes in hydration were not statistically significant (*p* = 0.12, *p* = 0.23, and *p* = 0.65, respectively). Erythema levels after one week (W1) of irritant application increased by 18% in zone I (*p* = 0.01), 13% in zone II (*p* = 0.03), and 20% in zone III (*p* = 0.009) compared to baseline (W0).

The application of a 5.0 wt.% anionic surfactant (SLES) solution during the irritation phase effectively induced controlled disruption of the stratum corneum lipid barrier, consistent with numerous literature reports on skin inflammation modeling [53]. The induction of barrier damage using the SLES solution has led to a rapid increase in TEWL by an average of 35 ± 2% (*p* < 0.05), clearly indicating intercellular lipid cement disintegration and disruption of the hydrolipidic film [53]. The slight concomitant increase in skin hydration (2–8%, *p* > 0.05) can be interpreted as a transient water-binding effect of the detergent’s hygroscopic molecules remaining in the stratum corneum, a phenomenon described in the literature as apparent hydration associated with acute irritation [54]. Simultaneously, the increase in erythema (13–20%, *p* < 0.05) confirmed activation of vasodilation in response to the irritant effect of SLES.

#### 3.4.2. Regeneration Phase (W1–W3)

**Figure 5:**
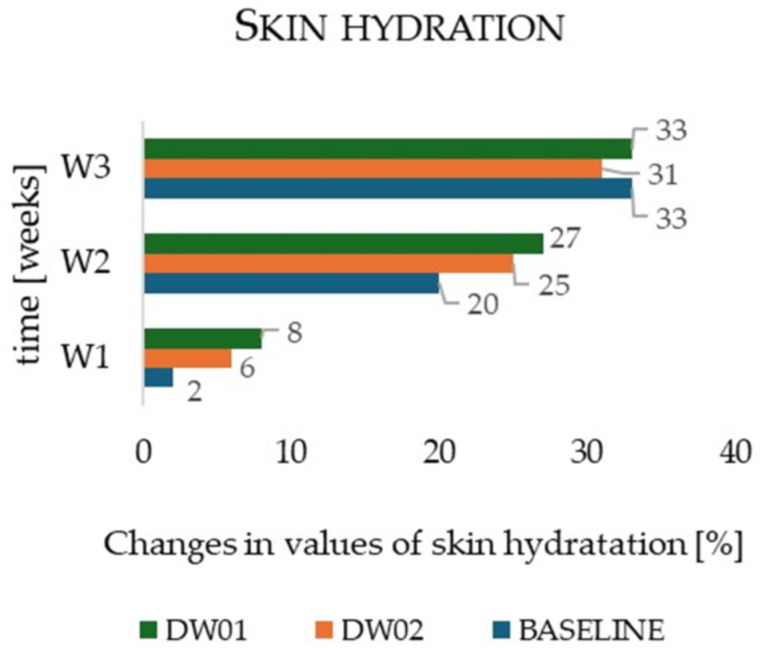
Changes in skin hydration determined for the cosmetics formulation DW01, DW02, and baseline during in vivo studies.

After completion of the 7-day irritant phase, a 3-week regenerative phase of forearm skin treatment was initiated according to the scheme presented in Figure 2 (Section 2.4.2). Following the first week of the regenerative program (W1-W2), stratum corneum hydration increased by 27% for the DW01 emulsion (zone I; *p* = 0.001), 25% for the DW02 emulsion (zone II; *p* = 0.002), and 20% for the control zone (zone III; *p* = 0.04) compared to the final day of the irritation phase (W1) (Figure 5). At this time point, hydration levels differed significantly among the three zones (*p* = 0.03 for zone I vs. II; *p* = 0.02 for zone I vs. III; *p* = 0.04 for zone II vs. III). After three weeks of product application (W3), skin hydration continued to increase, reaching +33% for DW01 (*p* = 0.001), +31% for DW02 (*p* = 0.001), and +33% for the control zone (*p* = 0.001), relative to W1. By the end of the study, no statistically significant differences in hydration were observed between the measurement zones (*p* > 0.05 for all comparisons).

**Figure 6:**
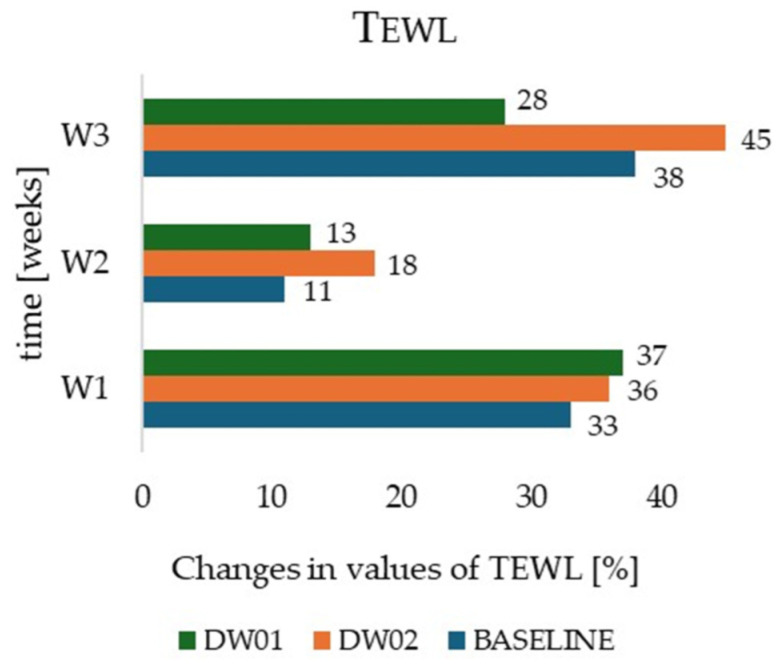
Changes in transepidermal water loss determined for the cosmetics formulation DW01, DW02, and baseline during in vivo studies.

In contrast, transepidermal water loss exhibited an increasing tendency during the regenerative phase. After the first week of application (W2), TEWL increased by 13% for DW01 and 18% for DW02 compared to values obtained at the end of the irritation phase (W1) (*p* < 0.05) (Figure 6). No significant differences in TEWL were detected between the measurement zones at this stage (*p* = 0.12 for zone I vs. II; *p* = 0.18 for zone I vs. III; *p* = 0.21 for zone II vs. III). After three weeks of treatment (W3), TEWL remained elevated, increasing by 28% for DW01 (*p* = 0.02), 45% for DW02 (*p* = 0.007) and 38% for the control zone (*p* = 0.01) relative to baseline values recorded at the start of the regenerative phase (W1). At the end of the study, statistically significant differences in TEWL were observed between the zones (*p* = 0.03 for zone I vs. II; *p* = 0.01 for zone I vs. III; *p* = 0.02 for zone II vs. III).

**Figure 7:**
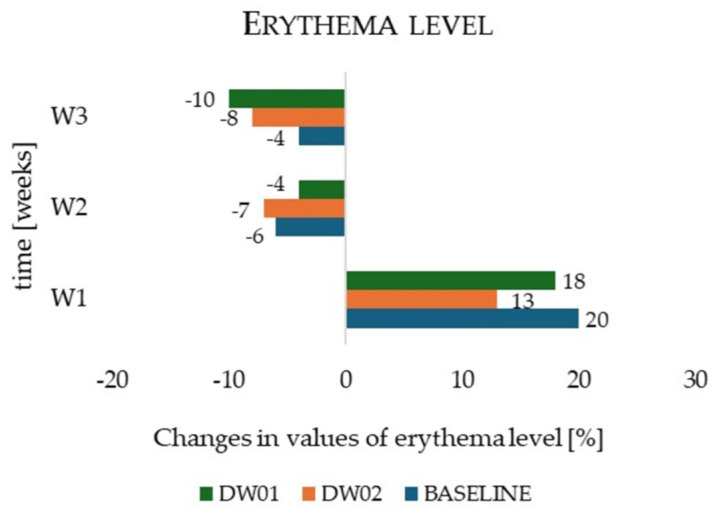
Changes in erythema level determined for the cosmetics formulation DW01, DW02, and baseline during in vivo studies.

Erythema levels showed a gradual reduction during the regenerative phase. After the first week of application (W2), erythema decreased by 4% for DW01, 7% for DW02, and 6% for the control zone relative to measurements obtained at the end of the irritation phase (W1) (Figure 7); however, these changes were not statistically significant, either within individual zones or between zones (*p* > 0.05). After three weeks of the regenerative program (W3), erythema continued to decline, with reductions of 10% for DW01 (*p* = 0.02), 8% for DW02 (*p* = 0.04), and 4% for the control zone (*p* = 0.06) compared to baseline values at W1. Comparative analysis revealed no significant difference between DW01 and DW02 (*p* = 0.07), whereas both emulsions differed significantly from the control zone (DW01 vs. control: *p* = 0.01; DW02 vs. control: *p* = 0.03).

The primary objective of the regenerative phase (W1–W3) was to evaluate the regenerative efficacy of the DW01 emulsion containing hop extract in comparison with the base emulsion DW02 and the untreated control zone (area without skincare application). Both DW01 and DW02 emulsions demonstrated moisturizing properties that increased with prolonged product use. However, the control zone also exhibited a sustained upward trend in skin hydration, reaching a 33% increase after extended observation (W3). In all three zones, the dynamics of hydration increased between weeks W2 and W3, followed by a similar pattern (*p* > 0.05). This observation may indicate a synergistic effect of the cosmetic base components and the bioactive substances present in the hop extract, as well as the intrinsic capacity of the epidermis for self-regeneration following SLES-induced irritation.

A key finding of the study concerned the influence of DW01 and DW02 formulations on the level of transepidermal water loss. The effects of the irritant phase, manifested as a marked increase in TEWL, were evident not only after the first week of SLES application (W1) but also persisted after three weeks of the regenerative program (W3). The paradoxical further increase in TEWL during the regenerative phase for both emulsions and the control zone may suggest that the 7-day SLES exposure induced profound alterations in the lipid structure of the epidermal barrier. In this context, barrier regeneration may require a longer time frame than the 21-day duration of the present study. Notably, DW01 exhibited the lowest rate of TEWL increase after three weeks of treatment (W3; +28%) compared with the base emulsion DW02 (+45%) and the control zone (+38%), which could support the hypothesis that hop extract may play a significant role in limiting secondary epidermal dehydration [55,56]. In this context, the regeneration products (DW01 and DW02) should not be interpreted as harmful, but rather as insufficiently occlusive and lipid-rich to fully restore and reseal the severely compromised hydrolipid barrier under conditions of such intensive prior irritation. Importantly, a marked increase in skin hydration, more than 30%, indicates effective humectant activity of the formulations. However, without a sufficiently strong occlusive component, the increased water content may not have been adequately retained within the stratum corneum, which could explain the persistent elevation in TEWL values. Therefore, these findings are interpreted as a valuable formulation insight rather than evidence of product inefficacy.

During the regenerative phase, DW01 also demonstrated the greatest efficacy in reducing erythema, lowering skin redness by 10% relative to the irritant phase. This effect was more pronounced than that observed in the control zone (4%, *p* = 0.01) and with the base emulsion DW02 (8%, *p* = 0.07). These results confirm the soothing properties of both formulations; however, the slight, although not statistically significant (*p* = 0.07), advantage of DW01 suggests an additional regenerative contribution of biologically active compounds present in the hop extract [42,57].

## 4. Conclusions

The antioxidant activity of biologically active compounds refers to their ability to neutralize reactive oxygen species (ROS), which are responsible for oxidative stress that can lead to the degradation of epidermal lipids and structural skin proteins [58,59]. Consequently, the use of substances with proven antioxidant activity is considered an important aspect of preventive skincare [60,61]. Studies have shown that *Humulus lupulus* L. is a valuable source of natural antioxidants, primarily due to its content of polyphenolic compounds [62], which exhibit well-established antioxidant activity (ABTS: 3.3–17.4 µmol TE/g; FRAP: 7.90–9.71 µmol TE/g; Folin–Ciocâlteu: 0.81–0.99 mg GAE/g). Notably, the values obtained in the present study fall within the ranges previously reported in the literature. After incorporation into the O/W emulsion (3.0 wt.% of HL extract), the antioxidant capacity of the finished product remained substantial (ABTS: 16.2 µmol TE/g; FRAP: 68.3 µmol TE/g; Folin–Ciocâlteu: 0.351 mg GAE/g), indicating functional stability and synergistic effects within the formulation matrix. It can be hypothesized that the HL extract has the potential to support skin protection against hypersensitivity by mitigating oxidative stress and inflammatory responses, thereby preventing irritation and inflammatory skin conditions [61,63]. Comparative analysis of the DW01 formulation enriched with HL extract and the base emulsion DW02 demonstrated differences in the epidermal barrier recovery and inflammatory response following controlled exposure to SLES. The HL formulation showed a lower TEWL increase (+28% vs. +45% for the base) and greater erythema reduction (−10% vs. −4% control), suggesting that its antioxidant activity may support attenuation of inflammatory responses. These effects indicate that hop extract may contribute to a faster soothing of skin irritation after exposure to aggressive detergents [35].

## Figures and Tables

**Table 1:** Chromatographic parameters used for the determination of hop compounds by HPLC.

Time [min]	Phase A [%]	Phase B [%]
0	80	20
3	80	20
33	25	75
37	0	100
40	0	100
47	80	20
50	80	20

**Table 2:** Retention times of hop-derived compounds determined by HPLC-DAD compared with literature data.

Compound	Retention Time [min]	Literature Range * [min]
humulinone	6.812	5–8
adhumulinone	8.195	7–9
iso-α-acids	8.737	8–12
iso-α-acids	9.793	8–12
xanthohumol	31.621	10–25
α-acids	38.638	35–40
isoxanthohumol	39.483	35–40
β-acids	39.684	38–42

* literature values were cited based on references [26,27,28,29].

**Table 3:** Results of accelerated aging tests for the DW01 emulsion (containing 3.0 wt.% of hop extract) after a 3-month storage period.

Parameter	4 °C	RT *	45 °C
Appearance (visual assessment)	homogeneous, no phase separation	homogeneous, no phase separation	homogeneous, no phase separation
Odor (sensory evaluation)	characteristic of raw materials used	characteristic of raw materials used	characteristic of raw materials used, but more intense
Color (visual assessment)	white to pale yellow	off-white	white to pale yellow
Consistency (visual assessment)	light with a rich sensation	light with a rich sensation	light with a rich sensation
pH	5.65 ± 0.01	5.61 ± 0.01	5.62 ± 0.02

* room temperature (15–25 °C/2Ph. Eur./WHO/GMP).

**Table 4:** Results of accelerated aging tests for the DW02 emulsion (without hop extract) after a 3-month storage period.

Parameter	4 °C	RT *	45 °C
Appearance (visual assessment)	homogeneous, no phase separation	homogeneous, no phase separation	homogeneous, no phase separation
Odor (sensory evaluation)	characteristic of raw materials used	characteristic of raw materials used	characteristic of raw materials used
Color (visual assessment)	white to pale beige	off-white	white to pale beige
Consistency (visual assessment)	light with a rich sensation	light with a rich sensation	light with a rich sensation
pH	5.56 ± 0.02	5.63 ± 0.02	5.65 ± 0.01

* room temperature (15–25 °C/2Ph. Eur./WHO/GMP).

**Table 5:** ABTS assay—absorbance parameters and TEAC * values for hop extract and xanthohumol.

	Concentration [wt.%]	Initial Absorbance [a.u.]	Final Absorbance [a.u.]	TEAC * [μmol TE/g]	Inhibition [%]
Hop extract (HL)	1.0	0.6923 ± 0.0004	0.5289 ± 0.0024	17.4	24
	2.0		0.3895 ± 0.0190	9.2	44
	3.0		0.2594 ± 0.0061	3.3	63
Xanthohumol	0.1	0.6305 ± 0.0002	0.5657 ± 0.0502	14.1	10
	0.2		0.4494 ± 0.0842	44.7	29
	0.3		0.3910 ± 0.0178	45.3	38

* Trolox Equivalent Antioxidant Capacity.

**Table 6:** Folin–Ciocâlteu assay—absorbance parameters and TPC * values for hop extract and xanthohumol.

	Concentration [wt.%]	Final Absorbance [a.u.]	TPC * [mg GAE/g]
Hop extract (HL)	1.0	0.0628 ± 0.0186	0.99
	2.0	0.0953 ± 0.0141	0.83
	3.0	0.1337 ± 0.0061	0.81
Xanthohumol	0.1	undetectable	---
	0.2	0.0934 ± 0.0049	8.08
	0.3	0.1895 ± 0.0101	11.93

* Total Phenolic Content.

**Table 7:** TPC values (mg GAE/g) for selected hop extracts—literature data.

Plant Material	TPC [mg GAE/g]	Comments	Literature Source
Hop (*Humulus lupulus* L.)	69.70–95.95	ethanol extract	[42]
	51.00–81.90	ethanol extract	[43]
	3.92–8.00	water–ethanol extract (1:4)	[44]
	0.51–6.60	methanol, ethanol extract	[45]

**Table 8:** FRAP assay—absorbance parameters and FRAP values for hop extract and xanthohumol.

	Concentration [wt.%]	Final Absorbance [a.u.]	FRAP [µmol TE/g]
Hop extract (HL)	1.0	0.2634 ± 0.0089	7.90
	2.0	0.3719 ± 0.0127	9.27
	3.0	0.4800 ± 0.0196	9.71
Xanthohumol	0.1	0.4157 ± 0.0107	228.3
	0.2	0.6710 ± 0.0906	239.3
	0.3	0.8240 ± 0.0616	209.5

**Table 9:** Comparison of the antioxidant activity of the designed cosmetic emulsion with literature data.

	Test Result	Literature Range	Literature Source
TEAC [µmol TE/g]	16.2	3.59–4.41 0.88–25.34	[50] [51]
FRAP [µmol TE/g]	68.3	*	---
TPC [mg GAE/g]	0.351	0.02–1.47	[52]

* literature data on the antioxidant activity of cosmetic creams are reported in various units, hindering direct comparison between studies.

## Data Availability

The original contributions presented in this study are included in the article. Further inquiries can be directed to the corresponding authors (the general procedure for the formation of emulsions is only available to non-professionals).

## Abbreviations

The following abbreviations are used in this manuscript:

ABTS	2,2′-azino-bis(3-ethylbenzothiazoline-6-sulfonic acid)
ABTS^+•^	ABTS Cation Radical
CPNP	Cosmetic Products Notification Portal
DSS	Dermal Sensitivity Syndrome
EC	European Commission
ESCOP	European Scientific Cooperative on Phytotherapy
FRAP	Ferric Reducing Antioxidant Power
GAE	Gallic Acid Equivalents
GMP	Good Manufacturing Practice
HL	*Humulus lupulus* extract
HMPC	Herbal Medicinal Product Committee
INCI	International Nomenclature of Cosmetic Ingredients
ISO	International Organization for Standardization
O/W	Oil in Water Emulsion
ROS	Reactive oxygen species
SLES	Sodium Laureth Sulfate
TAS2R	G protein-coupled bitter taste receptors
TE	Trolox Equivalent
TEAC	Trolox Equivalent Antioxidant Capacity
TEWL	Transepidermal Water Loss
TPC	Total Phenolic Content
TPTZ	2,4,6-tris(2-pyridyl)-s-triazine
UVB	Ultraviolet B
UV-Vis	Ultraviolet–Visible Spectroscopy
WHO	World Health Organization
W/O	Water in Oil Emulsion
